# Low preoperative hematocrit adversely affects short-term outcomes after arthroscopic rotator cuff repair

**DOI:** 10.1016/j.xrrt.2024.06.007

**Published:** 2024-07-20

**Authors:** Noah Kim, Kenny Ling, Katherine Wang, David E. Komatsu, Edward D. Wang

**Affiliations:** a Renaissance School of Medicine at Stony Brook University, Stony Brook, NY, USA; b Department of Orthopaedics, Stony Brook University, Stony Brook, NY, USA

**Keywords:** Rotator cuff, Arthroscopy, Anemia, Postoperative complications, Risk stratification, Shoulder

## Abstract

**Background:**

The purpose of this study was to investigate preoperative anemia as a risk factor for postoperative complications after arthroscopic rotator cuff repair (ARCR).

**Methods:**

Adult patients who underwent ARCR from 2015-2020 were identified in the American College of Surgeons National Surgical Quality Improvement Program database. Patients were grouped according to the following preoperative hematocrit levels: normal (male >39%, female >36%), mild anemia (male 33%-39%, female 33%-36%), and moderate to severe anemia (male and female ≤33%). Multivariable logistic regression analyses were performed to identify significant differences in 30-day postoperative complication rates.

**Results:**

Of the 21,836 patients identified, 19,726 (90.3%) patients had normal preoperative hematocrit, 1731 (7.9%) were mildly anemic, and 379 (1.7%) were moderate to severely anemic. After adjusting for significantly associated demographics and comorbidities, mild anemia was a significant predictor of any complication (odds ratio [OR] 1.436, *P* = .007), cardiac complications (OR 4.891, *P* = .002) sepsis-related complications (OR 4.760, *P* = .004), readmission (OR 1.585, *P* = .014), and nonhome discharge (OR 1.839, *P* = .006). Moderate to severe anemia was a significant predictor of any complication (OR 2.471, *P* < .001), readmission (OR 3.002, *P* < .001), and nonhome discharge (OR 3.211, *P* < .001).

**Conclusion:**

Preoperative anemia is a significant risk factor for postoperative complications within 30 days of ARCR.

For patients suffering from rotator cuff tears, arthroscopic rotator cuff repair (ARCR) can reduce pain and improve shoulder mobility and overall function.^18^^,^^21^ Compared to open repairs, ARCR has become the gold standard over the past few decades.^2^^,^^5^^,^^29^ Prior studies evaluating risk factors contributing to poor outcomes after ARCR found obesity, age, smoking, and diabetes to increase postoperative complication rates.^1^^,^^3^^,^^14^^,^^34^

Preoperative anemia, though well-established as a risk factor for poor postoperative outcomes in other orthopedic surgeries, has not been studied in the context of ARCR.^8^^,^^9^^,^^17^^,^^20^^,^^24^ As preoperative anemia may be a potential modifiable risk factor for adverse postoperative outcomes, it is worthwhile to explore this influence across various orthopedic surgeries. As such, the purpose of this study was to identify how varying degrees of preoperative anemia affected 30-day complication rates following ARCR.

## Methods

We queried the American College of Surgeons National Surgical Quality Improvement Program database (ACS NSQIP) for patients who underwent ARCR between 2015 and 2020. As all patient data in the ACS NSQIP database are deidentified, this study was exempt from our university’s institutional review board approval. The ACS NSQIP database collects patient data from over 706 community and academic hospitals. Data collection is performed by trained and certified clinical reviewers and is routinely audited by the ACS to ensure the highest quality data possible. Patients who underwent ARCR were identified by the current procedural terminology code 29827.

**Figure 1:**
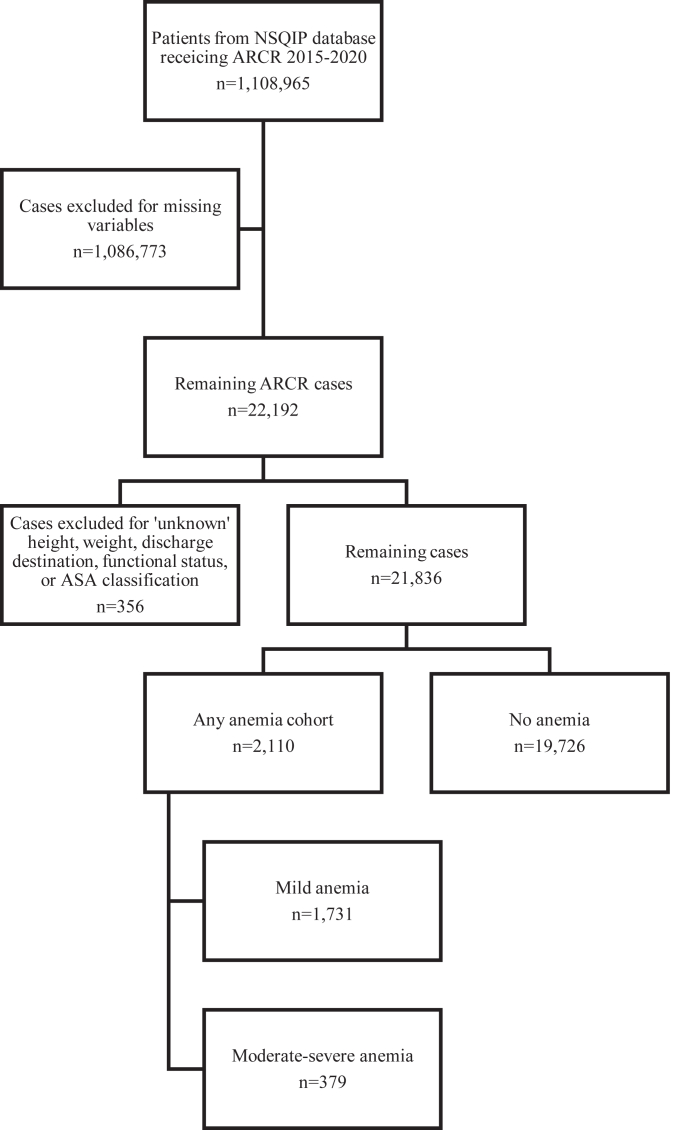
Strengthening the Reporting of Observational Studies in Epidemiology diagram with inclusion and exclusion criteria. *ARCR*, arthroscopic rotator cuff repair; *NSQIP*, National Surgical Quality Improvement Program.

Data on patient demographics and comorbidities were collected. Demographic variables included age and gender, and variables for preoperative comorbidities included obesity, diabetes, history of severe chronic obstructive pulmonary disease, congestive heart failure, smoking status within 1 year of surgery, hypertension requiring medication, bleeding disorders, and metastatic cancer. We also collected data on additional predictors, including American Society of Anesthesiologists (ASA) classification and functional status before surgery. Cases with missing data, or labeled ‘unknown’ height, weight, discharge destination, functional status, or ASA classification were excluded (1.6%, n = 356,) (Fig. 1). Patients under the age of 18 years were also excluded.

Per the World Health Organization’s sex-based criteria for anemia, patients were considered anemic if preoperative hematocrit measured within 30 days was ≤36% for females and ≤39% for males.^28^ Hematocrit cutoffs for mild and moderate to severe anemia were defined according to those used in previous studies.^9^^,^^30^ Mild anemia was defined as hematocrit between 33%-36% in females and 33%-39% in males, while moderate to severe anemia was defined as hematocrit ≤33% for both sexes. Patients with anemia were grouped into 4 cohorts for analysis: patients without anemia, patients with mild anemia, patients with moderate to severe anemia, and a pooled group of patients with any degree of anemia.

We identified 21,836 patients who underwent ARCR between 2015 and 2020. Of these patients, 19,726 (90.3%) patients had normal preoperative hematocrit and 2110 (9.7%) patients had anemia. Of the anemic patients, 1731 (82.0%) had mild anemia and 379 (18.0%) had moderate to severe anemia.

Study outcomes were defined by the 30-day postoperative complications recorded in the ACS NSQIP database and grouped by complication type. Medical complications included respiratory complications (reintubation, ventilation requirements for >48 hours, pneumonia), cardiac complications (myocardial infarction, cardiac arrest requiring cardiopulmonary resuscitation), sepsis (sepsis, septic shock), thromboembolic events (deep vein thrombosis, pulmonary embolism), renal complications (progressive renal insufficiency, acute renal failure), urinary tract infection, and stroke/cerebrovascular accident. Surgical complications included superficial incisional surgical site infection (SSI), deep incisional SSI, organ/space SSI, and wound dehiscence. Additional complications included postoperative bleeding requiring transfusion, readmission, reoperation, and nonhome discharge.

All statistics were performed with SPSS version 29.0 (IBM Corp., Armonk, NY, USA). Bivariate analyses were conducted to identify differences within anemia cohorts as well as to identify any variables significantly associated with outcomes. Pearson chi-square and Fisher’s exact tests were used for comparison of categorical variables. Bivariate analysis with Bonferroni-corrected pairwise chi-square tests was conducted for multilevel categorical variables (results not shown). Complication rates between anemia cohorts were compared before multivariable logistic regression analysis. Multivariable binomial logistic regression, adjusting for predictors found to be significantly associated with outcomes, was performed to characterize associations between varying degrees of anemia and postoperative complications. Complication types with either 0 occurrences in any cohort, or less than 5 occurrences in both cohorts involved in a comparison were excluded from multivariable logistic regression analysis. Statistical significance was set to *P* < .05 for both bivariate tests and regression analyses.

## Results

Compared to the normal hematocrit cohort, a greater proportion of patients in the any anemia cohort were above the age of 65, women, morbidly obese, and functionally dependent. A greater proportion of anemic patients had hypertension, diabetes, severe chronic obstructive pulmonary disease, congestive heart failure, bleeding disorders, metastatic cancer, and belonged to ASA class ≥3 (Table I).

**Table I:** Patient demographics/comorbidities in patients with and without mild or moderate to severe preoperative anemia who underwent arthroscopic rotator cuff repair.

Characteristic	Preoperative hematocrit											
	Normal		Any anemia			Mild anemia			Moderate to severe anemia			Mild vs. moderate to severe
	Count	Percent	Count	Percent	*P* value	Count	Percent	*P* value	Count	Percent	*P* value	*P* value
Total	19,726	100.0%	2110	100.0%		1731	100.0%		379	100.0%		
Age					**<.001**			**<.001**			**.005**	.159
18-40	532	2.7%	33	1.6%		25	1.4%		8	2.1%		
40-65	12434	63.1%	1137	54.0%		929	53.8%		208	55.0%		
65-75	5777	29.3%	730	34.7%		594	34.4%		136	36.0%		
75+	975	4.9%	206	9.8%		180	10.4%		26	6.9%		
Sex					**.008**			.099			**<.001**	**<.001**
Female	8506	43.1%	973	46.1%		711	41.1%		262	69.1%		
Male	11,220	56.9%	1137	53.9%		1020	58.9%		117	30.9%		
Body mass index (kg/m^2^)					**.006**			**.018**			.057	.248
18.5-25	2775	14.1%	329	15.6%		279	16.1%		50	13.2%		
<18.5	79	0.4%	12	0.6%		9	0.5%		3	0.8%		
25-30	6588	33.4%	707	33.5%		589	34.0%		118	31.1%		
30-35	5520	28.0%	528	25.0%		432	25.0%		96	25.3%		
35-40	2786	14.1%	286	13.6%		229	13.2%		57	15.0%		
40+	1978	10.0%	248	11.8%		193	11.1%		55	14.5%		
Functional status					**<.001**			**<.001**			**.029**	1.000
Independent	19,640	99.6%	2083	98.7%		1709	98.7%		374	98.7%		
Dependent	86	0.4%	27	1.3%		22	1.3%		5	1.3%		
Current smoker					**<.001**			**.005**			**.017**	.299
No	16,774	85.0%	1854	87.9%		1515	87.5%		339	89.4%		
Yes	2952	15.0%	256	12.1%		216	12.5%		40	10.6%		
Hypertension					**<.001**			**<.001**			**<.001**	.678
No	9762	49.5%	704	33.4%		581	33.6%		123	32.5%		
Yes	9964	50.5%	1406	66.6%		1150	66.4%		256	67.5%		
Diabetes					**<.001**			**<.001**			**<.001**	.277
No	15,995	81.1%	1414	67.0%		1151	66.5%		263	69.4%		
Yes	3731	18.9%	696	33.0%		580	33.5%		116	30.6%		
COPD					**.045**			.102			.176	.644
No	19,043	96.5%	2019	95.7%		1658	95.8%		361	95.3%		
Yes	683	3.5%	91	4.3%		73	4.2%		18	4.7%		
CHF					**<.001**			**.001**			**<.001**	**.038**
No	19,700	99.9%	2095	99.3%		1722	99.5%		373	98.4%		
Yes	26	0.1%	15	0.7%		9	0.5%		6	1.6%		
Bleeding disorders					**<.001**			**<.001**			**<.001**	.308
No	19,395	98.3%	2029	96.2%		1668	96.4%		361	95.3%		
Yes	331	1.7%	81	3.8%		63	3.6%		18	4.7%		
Disseminated cancer					**.015**			.098			**<.024**	.221
No	19,715	99.9%	2105	99.8%		1728	99.8%		377	99.5%		
Yes	11	0.1%	5	0.2%		3	0.2%		2	0.5%		
ASA classification					**<.001**			**<.001**			**<.001**	.594
1-2	11,695	59.3%	872	41.3%		720	41.6%		152	40.1%		
≥3	8031	40.7%	1238	58.7%		1011	58.4%		227	59.9%		

*ASA*, American Society of Anesthesiologists; *COPD*, chronic obstructive pulmonary disease; *CHF*, congestive heart failure.

Bold *P* values indicate statistical significance with *P* < .05.

Relative to the nonanemic cohort, the rate of any complication was nearly doubled in the mild anemia cohort (4.1% vs. 2.3%, *P* < .001), and tripled in the moderate to severe anemia group (6.9% vs. 2.3%, *P* < .001). The rate of any complication of the moderate to severe anemia cohort was also significantly different from mild anemia (*P* = .020). In comparison to the nonanemic cohort, those with mild anemia had a higher rate of overall medical complications (1.2% vs. 0.7%, *P* = .034), including respiratory complications (0.4% vs. 0.2%, *P* = .034), cardiac complications (0.4% vs. 0.1%, *P* < .001), sepsis-related complications (0.3% vs. 0.1%, *P* = .002), and renal complications (0.3% vs. 0.0%, *P* < .001). Compared to nonanemic patients, patients with mild anemia also had higher rates of readmission (2.1% vs. 1.0%, *P* < .001) and nonhome discharge (1.6% vs. 0.7%, *P* < .001) (Table II). Compared to the nonanemic cohort, moderate to severe anemia was associated with higher rates of readmission (3.7% vs. 1.0%, *P* < .001) and nonhome discharge (2.9% vs. 0.7% *P* < .001).

**Table II:** Bivariate analysis of 30-day postoperative complications in patients with preoperative anemia.

Complication	Preoperative hematocrit											
	Normal (N = 19,726)		Any anemia (N = 2110)			Mild anemia (N = 1731)			Moderate to severe anemia (N = 379)			Mild vs. moderate to severe
	Count	Percent	Count	Percent	*P* value	Count	Percent	*P* value	Count	Percent	*P* value	*P* value
Any complication	19,263	2.3%	97	4.6%	**<.001**	71	4.1%	**<.001**	26	6.9%	**<.001**	**.020**
Medical complication	147	0.7%	27	1.3%	**.009**	21	1.2%	**.034**	6	1.6%	.070	.612
Respiratory complication	32	0.2%	9	0.4%	**.015**	7	0.4%	**.034**	2	0.5%	.134	.668
Reintubation	3	0.0%	4	0.2%	**.002**	3	0.2%	**.009**	1	0.3%	.073	.547
Ventilator >48 h	3	0.0%	2	0.1%	.077	2	0.1%	.055	0	0.0%	-	-
Pneumonia	28	0.1%	6	0.3%	.136	5	0.3%	.185	1	0.3%	.424	1.000
Cardiac complication	11	0.1%	7	0.3%	**<.001**	7	0.4%	**<.001**	0	0.0%	-	-
Myocardial infarction	9	0.0%	7	0.3%	**<.001**	7	0.4%	**<.001**	0	0.0%	-	-
Cardiac arrest	2	0.0%	0	0.0%	-	0	0.0%	-	0	0.0%	-	-
Sepsis complication	11	0.1%	7	0.3%	**<.001**	6	0.3%	**.002**	1	0.3%	.204	1.000
Sepsis	11	0.1%	6	0.3%	**.004**	5	0.3%	**.007**	1	0.3%	.204	1.000
Septic shock	0	0.0%	1	0.0%	-	1	0.1%	-	0	0.0%	-	-
DVT/PE	48	0.2%	5	0.2%	.955	4	0.2%	1.000	1	0.3%	.607	1.000
Deep vein thrombosis	31	0.2%	2	0.1%	.766	1	0.1%	.514	1	0.3%	.456	.327
Pulmonary embolism	22	0.1%	3	0.1%	.729	3	0.2%	.450	0	0.0%	-	-
Renal complication	1	0.0%	5	0.2%	**<.001**	5	0.3%	**<.001**	0	0.0%	-	-
Progressive renal insufficiency	1	0.0%	3	0.1%	**.003**	3	0.2%	**.002**	0	0.0%	-	-
Acute renal failure	0	0.0%	2	0.1%	-	2	0.1%	-	0	0.0%	-	-
Urinary tract infection	49	0.2%	4	0.2%	.602	2	0.1%	.436	2	0.5%	.250	.150
Stroke/CVA	6	0.0%	1	0.0%	.509	1	0.1%	.445	0	0.0%	-	-
Surgical complication	47	0.2%	4	0.2%	.815	4	0.2%	1.000	0	0.0%	-	-
Superficial incisional SSI	25	0.1%	2	0.1%	1.000	2	0.1%	1.000	0	0.0%	-	-
Deep incisional SSI	10	0.1%	1	0.0%	1.000	1	0.1%	.604	0	0.0%	-	-
Organ/Space SSI	9	0.0%	1	0.0%	1.000	1	0.1%	.569	0	0.0%	-	-
Wound dehiscence	5	0.0%	0	0.0%	-	0	0.0%	-	0	0.0%	-	-
Postoperative transfusion	1	0.0%	1	0.0%	.184	1	0.1%	.155	0	0.0%	-	-
Readmission	204	1.0%	51	2.4%	**<.001**	37	2.1%	**<.001**	14	3.7%	**<.001**	.074
Reoperation	53	0.3%	9	0.4%	.195	9	0.5%	.062	0	0.0%	-	-
Nonhome discharge	132	0.7%	39	1.8%	**<.001**	28	1.6%	**<.001**	11	2.9%	**<.001**	.093

*CVA*, cerebrovascular accident; *SSI*, surgical site infection; *DVT*, deep vein thrombosis; *PE*, pulmonary embolism; *SSI*, surgical site infection.

Bold *P* values indicate statistical significance with *P* < .05.

Multivariable regression analysis, after adjusting for confounders identified in Table I, found that any anemia increased the odds of at least 1 postoperative complication (OR 1.605, *P* < .001). Mild anemia was found to be a significant predictor of any complication (OR 1.436, *P* = .007), cardiac complications (OR 4.891, *P* = .002), sepsis-related complications (OR 4.760, *P* = .004), readmission (OR 1.585, *P* = .014), and nonhome discharge (OR 1.839, *P* = .006) (Table III). Stepwise increases in the odds of any complication (OR 2.471, *P* < .001), readmission (OR 3.002, *P* < .001), and nonhome discharge (OR 3.211, *P* < .001) were observed in the moderate to severe anemia compared to the nonanemic group. Patients with moderate to severe anemia, adjusting for confounders, were nearly twice as likely to experience any complication relative to mild anemia (OR 1.816, *P* = .022).

**Table III:** Multivariable analysis of 30-day postoperative complications in patients with preoperative anemia, adjusted for significantly associated patient demographics and comorbidities.

Complications	Normal vs. any anemia			Normal vs. mild			Normal vs. moderate to severe			Mild vs. moderate to severe		
	OR	95% CI	*P* value	OR	95% CI	*P* value	OR	95% CI	*P* value	OR	95% CI	*P* value
Any complication	1.605	(1.269-2.029)	**<.001**	1.436	(1.102-1.872)	**.007**	2.471	(1.606-3.800)	**<.001**	1.816	(1.091-3.022)	**.022**
Medical complication	1.356	(0.882-2.084)	.165	1.324	(0.825-2.126)	.245	1.491	(0.620-3.586)	.372	1.416	(0.521-3.846)	.495
Respiratory complication	2.046	(0.943-4.441)	.070	1.850	(0.786-4.353)	.159	2.837	(0.654-12.302)	.164	1.557	(0.271-8.951)	.620
Cardiac complication	4.084	(1.528-10.919)	**.005**	4.891	(1.822-13.128)	**.002**	-	-	-	-	-	-
Sepsis complication	4.031	(1.425-11.405)	**.009**	4.760	(1.657-13.671)	**.004**	1.169	(0.053-25.900)	.921	-	-	-
DVT/PE	1.003	(0.393-2.561)	.995	0.975	(0.346-2.748)	.962	1.132	(0.154-8.337)	.903	-	-	-
Urinary tract infection	0.566	(0.201-1.598)	.283	0.361	(0.086-1.507)	.162	1.239	(0.290-5.303)	.773	-	-	-
Stroke/CVA	1.232	(0.141-10.791)	.850	1.605	(0.182-14.156)	.670	-	-	-	-	-	-
Surgical complication	0.908	(0.323-2.558)	.855	1.078	(0.382-3.039)	.887	-	-	-	-	-	-
Superficial incisional SSI	0.847	(0.197-3.641)	.823	1.021	(0.237-4.394)	.978	-	-	-	-	-	-
Deep incisional SSI	1.077	(0.133-8.746)	.945	1.256	(0.154-10.231)	.831	-	-	-	-	-	-
Organ/Space SSI	1.232	(0.151-10.052)	.845	1.374	(0.168-11.203)	.767	-	-	-	-	-	-
Readmission	1.810	(1.310-2.499)	**<.001**	1.585	(1.100-2.286)	**.014**	3.002	(1.691-5.332)	**<.001**	1.806	(0.930-3.507)	.081
Reoperation	1.602	(0.774-3.319)	.205	1.931	(0.931-4.005)	.077	-	-	-	-	-	-
Nonhome discharge	2.071	(1.403-3.057)	**<.001**	1.839	(1.187-2.848)	**.006**	3.211	(1.606-6.419)	**<.001**	1.756	(0.799-3.863)	.161

*DVT*, deep vein thrombosis; *PE*, pulmonary embolism; *CVA*, cerebrovascular accident; *SSI*, surgical site infection; *OR*, odds ratio; *CI*, confidence interval.

Bold *P* values indicate statistical significance with *P* < .05.

## Discussion

In this retrospective study of 21,836 patients from a large national database, preoperative anemia was associated with higher rates of 30-day postoperative outcomes after ARCR. After adjusting for the effects of other significant predictors, preoperative anemia was found to increase the odds of cardiac complications, sepsis or septic shock, readmission, and nonhome discharge. We also demonstrated that the severity of preoperative anemia corresponded to increased risk of adverse outcomes.

Preoperative anemia is a well-characterized risk factor for adverse outcomes following orthopedic surgery. In the setting of total hip arthroplasty, total knee arthroplasty, total shoulder arthroplasty (TSA), spine surgery, and orthopedic trauma, preoperative anemia has been associated with postoperative blood transfusions, medical complications, surgical site and periprosthetic joint infections, reoperation, increased mortality, nonhome discharge, and readmission.^7^, ^8^, ^9^, ^10^^,^^13^^,^^16^^,^^17^^,^^20^^,^^22^^,^^24^^,^^25^^,^^27^^,^^30^^,^^31^ Our results were generally consistent with these prior studies. The prevalence of anemia within our study population (9.7%) was similar to rates between 8.7%-19.2% described in large database studies involving total hip arthroplasty, total knee arthroplasty, TSA, and anterior cervical discectomy and fusion surgeries.^7^^,^^9^^,^^12^^,^^13^^,^^20^^,^^30^ Anemic patients in our cohort were also older and shared a greater burden of comorbidities relative to nonanemic patients. This was consistent with epidemiologic and retrospective studies reporting higher medical comorbidity prevalence, mortality rate, functional dependence, and frailty in both surgical and nonsurgical anemic populations.^4^^,^^6^^,^^7^^,^^30^

While the greater comorbidity burden may confound the observed relationship between preoperative anemia and greater complications, our results suggest otherwise. Comparisons between mild and moderate to severe anemia groups across all predictor variables revealed minimal significant differences. In the multivariable regression analysis, however, the odds of a complication were nearly doubled for moderate to severe anemia vs. mild anemia for any complication and for complication categories where both differed significantly from the normal hematocrit group. These results are consistent with the few existent studies evaluating the effect of anemia severity on odds of complications after orthopedic surgery, which found a significant increase in the odds of any complication with increasing severity of anemia from mild (OR 1.46-2.74) to severe (1.99-7.96).^7^^,^^9^^,^^13^^,^^30^ In direct comparisons between moderate to severe and mild anemia, the odds of complications for moderate to severe anemia were similarly nearly twice that of the mild anemia group, though not all differences were significant. While multimorbidity prevalence (eg, comorbidity index) outside of ASA status was not specifically assessed between groups, the observed differences in odds ratios between groups with similar comorbidity rates suggest that low preoperative hematocrit may exert an independent influence on complication rates.

In the context of anemia and arthroscopic shoulder surgery, a study by Hill et al included preoperative hematocrit in their analysis of risk factors for 30-day readmission following any arthroscopic shoulder procedure. They identified a significant difference in preoperative hematocrit between readmitted (hematocrit 40.6%) and nonreadmitted patients (hematocrit 41.7%) in bivariate analysis.^11^ However, they did not stratify severity of anemia by hematocrit levels and the difference observed in bivariate analysis was not significant after adjusting for confounders in their multivariable regression analysis.^11^

In the setting of nonarthroscopic shoulder surgery, our study shares similarities with recent large retrospective database studies examining the effects of varying degrees of preoperative anemia on outcomes after reverse and anatomic TSA. Consistent with Kashanchi et al, we found mild anemia increased the odds of any 30-day postoperative complication, nonhome discharge, and readmission. We also found moderate to severe anemia to increase the odds of nonhome discharge.^13^ Consistent with Wang et al, we found anemia to be associated with an increased risk of any complication and cardiac complications.^30^

In general, complications after ARCR are uncommon, with several studies reporting rates of 1.1%-1.4%.^3^^,^^11^^,^^14^^,^^19^^,^^23^^,^^26^ Of the described complications, cardiac complications in particular have a more direct and mechanistic association with preoperative anemia, especially in the elderly population.^32^ While we found increased rates of overall cardiac complications for mild anemia, severe anemia displayed no association with cardiac complications, contrary to expectations. It is possible that the smaller group size in the severe anemia cohort coupled with the low overall incidence of complications increased the likelihood of failing to detect any significant difference in cardiac complications that may have existed.

The retrospective nonmatched cohort design of this study limits the applicability of our findings. Specifically, we observed a higher rate of comorbidities in the anemia groups compared to the normal hematocrit groups which may have confounded results. While preoperative anemia in the present study was defined by low preoperative hematocrit, future inquiries should accommodate additional metrics such as hemoglobin, as well as additional hematologic criterion of anemia. As the ACS NSQIP database only records 30-day postoperative complications, long-term and functional outcomes are unknown. The ACS NSQIP database also does not collect data on surgeries performed at non–hospital-affiliated ambulatory surgery centers. In addition, as ARCR are frequently performed outpatient, our patient population may not be completely representative.^15^^,^^33^ Finally, the details of this analysis were limited by lack of available data in the ACS NSQIP database pertaining to the etiology of anemia. Aside from preoperative transfusion within 72 hours of surgery, no other data on the preoperative optimization status of anemic patients were available.

## Conclusion

Mild preoperative anemia, as indicated by low preoperative hematocrit, was found to be a risk factor for cardiac complications, sepsis, readmission, and nonhome discharge following ARCR. Moderate to severe anemia was found to be a risk factor for readmission and nonhome discharge. We also identified a stepwise increase in the odds of nonhome discharge and readmission in moderate to severe anemia compared to mild anemia.

## Disclaimers:

Funding: This research received no specific grant from any funding agency in the public, commercial, or not-for-profit sectors.

Conflicts of interest: The authors, their immediate families, and any research foundations with which they are affiliated have not received any financial payments or other benefits from any commercial entity related to the subject of this article.
